# Progress in Oral Vaccination against Tuberculosis in Its Main Wildlife Reservoir in Iberia, the Eurasian Wild Boar

**DOI:** 10.1155/2012/978501

**Published:** 2012-07-10

**Authors:** Beatriz Beltrán-Beck, Cristina Ballesteros, Joaquín Vicente, José de la Fuente, Christian Gortázar

**Affiliations:** ^1^Instituto de Investigación de Recursos Cinegéticos, IREC (CSIC-UCLM-JCCM), Ronda de Toledo s/n, 13071 Ciudad Real, Spain; ^2^Department of Veterinary Pathobiology, Center for Veterinary Health Sciences, Oklahoma State University, Stillwater, OK 74078, USA

## Abstract

Eurasian wild boar (*Sus scrofa*) is the main wildlife reservoir for tuberculosis (TB) in Iberia. This review summarizes the current knowledge on wild boar vaccination including aspects of bait design, delivery and field deployment success; wild boar response to vaccination with Bacillus Calmette-Guérin (BCG) and inactivated Mycobacterium bovis; and wild boar vaccination biosafety issues as well as prospects on future research. Oral vaccination with BCG in captive wild boar has shown to be safe with significant levels of protection against challenge with virulent *M. bovis*. An oral vaccination with a new heat-killed *M. bovis* vaccine conferred a protection similar to BCG. The study of host-pathogen interactions identified biomarkers of resistance/susceptibility to tuberculosis in wild boar such as complement component 3 (C3) and methylmalonyl coenzyme A mutase (MUT) that were used for vaccine development. Finally, specific delivery systems were developed for bait-containing vaccines to target different age groups. Ongoing research includes laboratory experiments combining live and heat-killed vaccines and the first field trial for TB control in wild boar.

## 1. Introduction

Total eradication of an infectious agent shared between wild and domestic animals is almost impossible if a native wildlife host is able to serve as a natural reservoir of the pathogen [1–3]. Tuberculosis (TB) is a chronic disease caused by infection with *Mycobacterium bovis* and closely related members of the *Mycobacterium tuberculosis* complex (MTC). TB affects not only cattle but also a range of other livestock, companion animals, and wild animals. Humans are also susceptible; hence control of the risks of zoonotic infection is a driver for disease control in animal hosts. As TB prevalence has been reduced in livestock, the relative epidemiological and socio-economic importance of wildlife reservoirs has increased and there is a corresponding need for disease management strategies to reflect this effect [4].

Disease control through vaccination of wildlife reservoirs has advantages over other approaches. When dealing with disease maintenance by native wildlife, vaccination—as opposed to culling—is a nondestructive method of controlling disease that is more acceptable to the public [5, 6]. The primary goal of a wildlife vaccine would be to reduce the prevalence of infection in the wildlife reservoir or to change the expression of the disease and limit the rate of *M. bovis* excretion [7]. Indeed, vaccination is nowadays explored as an option for TB control in all major wildlife reservoir hosts such as the brushtail possum (*Trichosurus vulpecula*) in New Zealand, Eurasian badger (*Meles meles*) the United Kingdom and the Republic of Ireland, and white-tailed deer (*Odocoileus virginianus*) in the USA, among others [8]. 

In Mediterranean habitats of the south-western Iberian Peninsula, the abundant and widespread native Eurasian wild boar (*Sus scofa*) is an important driver in *M. bovis* epidemiology [9], thus the need for TB control in this species. Since uninfected 2–4-month-old wild boar piglets are the preferred age class for vaccination [10], an oral delivery system that targets piglets is needed for field application of oral tuberculosis vaccines in wild boar. After briefly introducing the role of the wild boar in tuberculosis (TB) epidemiology and the options for TB control, this review summarizes the current knowledge on wild boar vaccination including aspects of bait design, delivery, and field deployment success; wild boar response to vaccination with BCG and inactivated *Mycobacterium bovis*; wild boar vaccination biosafety issues as well as prospects on future research. 

## 2. Tuberculosis in Eurasian Wild Boar

The Eurasian wild boar is the ancestor of the domestic pig. It is native to Eurasia and the north of Africa and has been introduced, pure or crossbred, to many other regions worldwide. TB is one of the main infections shared between wild boar and other wild and domestic animals [4]. TB has a complex epidemiology involving multiple hosts and is influenced by climate, habitat, and management factors. Consequently, the role of wild and domestic hosts in TB epidemiology varies among regions [11]. 

Despite the success of compulsory test and slaughter campaigns in cattle, bovine TB (bTB) is still present in the Iberian Peninsula, and the role of wildlife reservoirs is increasingly recognized [4, 11, 12]. In Mediterranean habitats of southern Portugal and south-western Spain, MTC transmission occurs among three wild ungulate species, wild boar, red deer (*Cervus elaphus)*, as well as fallow deer (*Dama dama*), cattle, and to a lesser extent other domestic and wild animals such as goats, pigs, and Eurasian badgers [4, 13, 14]. 

Nevertheless, there is consensus in defining the wild boar as the single most important TB reservoir host in this region [9, 13, 15–17]. Extremely high densities and high contact rates within social groups and at waterholes or focal food sources might contribute to the high TB prevalence, often over 40% prevalence [12, 13, 16, 18–20]. Wild boar experience higher levels of exposure than deer [18] and are at greater infection risk as a result of feeding on tuberculous carrion [13]. Finally, wild boar are more likely than deer to push their way under fences, facilitating contact with livestock [12]. Work on TB time trends in Iberian wild boar has shown a stable prevalence with local variability, as well as an apparent expansion of the infection to new sites [21]. Moreover, wild boar TB has already been described in at least ten European countries (Bulgaria, Croatia, France, Germany, Hungary, Italy, Portugal, Slovakia, Spain, and the UK), and evidence as wildlife reservoirs is growing beyond the peculiar high-density and intense-management systems of south-central Iberia [4]. 

Naturally, MTC-infected wild boar show visible lesions in over 80% of the cases and only microscopic lesions in another 9% [22]. The distribution of lesions is generalized in two thirds of the cases, meaning that they are evident in more than one anatomic region. The mandibular lymph node (mLN) is the most frequently affected organ while large generalized and lung lesions are more frequent among 1 to 2 year old subadults, which have the highest potential to excrete mycobacteria and can die due to the disease [22]. Prevalence increases with age, < 6 month-old piglets showing a mean of 10% prevalence [15]. 

In contrast to wild boar, the role of feral pigs as MTC reservoirs is questioned [23–25]. However, domestic pigs and free-ranging *Sus scrofa* are MTC hosts in southwestern Spain [26], the Mediterranean islands of Corsica, Sardinia and Sicily [27–29], and the Hawaiian island of Molokai [30]. In Argentina, a wild-boar-derived *M. bovis* strain proved more pathogenic than the reference cattle-derived strain in a cattle challenge model [31]. Hence, the role of suids in the maintenance of MTC deserves more attention worldwide.

## 3. Options for TB Control in Wild Boar

The first requisite for any disease control in wildlife is establishing a proper monitoring scheme [32]. Then, actions towards disease control can be critically assessed. TB control in wildlife reservoir hosts can eventually be achieved by different means, including (1) the improvement of biosecurity and hygiene, (2) population control through random or selective culling or through habitat management, and (3) vaccination. Ideally, tools from all three fields should be combined in an integrated control strategy. 

In this context, wildlife vaccination to reduce MTC infection prevalence emerges as a valuable alternative or complementary tool in TB control [43]. Capturing wild animals to vaccinate them individually is expensive, time consuming, and difficult [44]. Therefore, the most feasible approach to deliver vaccines to wildlife is the use of oral baits. 

Oral vaccination against rabies was the first successful attempt to control a disease in wildlife through vaccination [45]. Thus, oral bait vaccination has also been considered for controlling other diseases such as classical swine fever in wild boar in Europe [33, 46] or TB in several wildlife hosts worldwide [47]. 

## 4. Bait Design, Selective Delivery, and Field Deployment Success

The effective and efficient field vaccination of wildlife requires the development of baits that are stable under field conditions, safe for target and nontarget species as well as the environment, and effective in reaching the target species [48–50]. A wide variety of baits have been developed in order to deliver pharmaceuticals to wild species. Lipid-based baits have been tested to deliver BCG vaccine against TB in wild animal species that act as reservoirs hosts such as badgers in United Kingdom and Republic of Ireland [44], possums in New Zealand [51–53], and while-tailed deer in USA [54].

For free-ranging *Sus scrofa*, three different baits have been developed and used for the oral delivery of vaccines and pharmaceuticals (Table 1). All of them are made with a cereal-based matrix containing a capsule or blister to deliver the vaccine or pharmaceutical. The palatable ingredients used for the bait matrix composition stimulate chewing to open the capsules contained in the baits and releasing their content inside the oral cavity [36].

For TB vaccination, wild boar piglets (rather than already infected adults) are the main target [10]. If BCG was used, accidental bait uptake by cattle needs to be avoided [55]. Hence, purpose-designed baits and oral delivery systems selective for piglets are needed. The three baits developed for free-ranging *Sus scrofa* have been found to be highly attractive and readily ingested by animals [33, 36, 37, 39, 40]. However, both Spanish and PIGOUT baits have been found to be not target-specific enough in those areas where other wildlife species can also have access to the baits [10, 41, 42]. No data concerning target specificity of Riemser baits has been published.

Field assessment of the proportion of target and nontarget individuals that consume baits is crucial to evaluate the success of a baiting campaign. Therefore, marking agents are incorporated into baits to enable identification of consuming individuals [56]. Iophenoxic acid (*α*-ethyl-2-hydroxy-2,4,6-triiodebenzenepropanoic acid) and its derivatives have been used successfully to investigate baits and baiting strategies to deliver orally vaccines, contraceptives, and toxicants [41, 57–61], since they bind to protein in the blood plasma and elevate the protein-bound iodine of animals which consume them. So, these markers can be detected in the serum of animals consuming IPA-marked baits for a long time after their ingestion [62]. In the case of wild boar, Ballesteros et al. [63] found that ethyl and propyl-iophenoxic acids could be detected in animal serum up to 18 months after their consumption when doses of 5 and 15 mg/kg were delivered.

**Figure 1:**
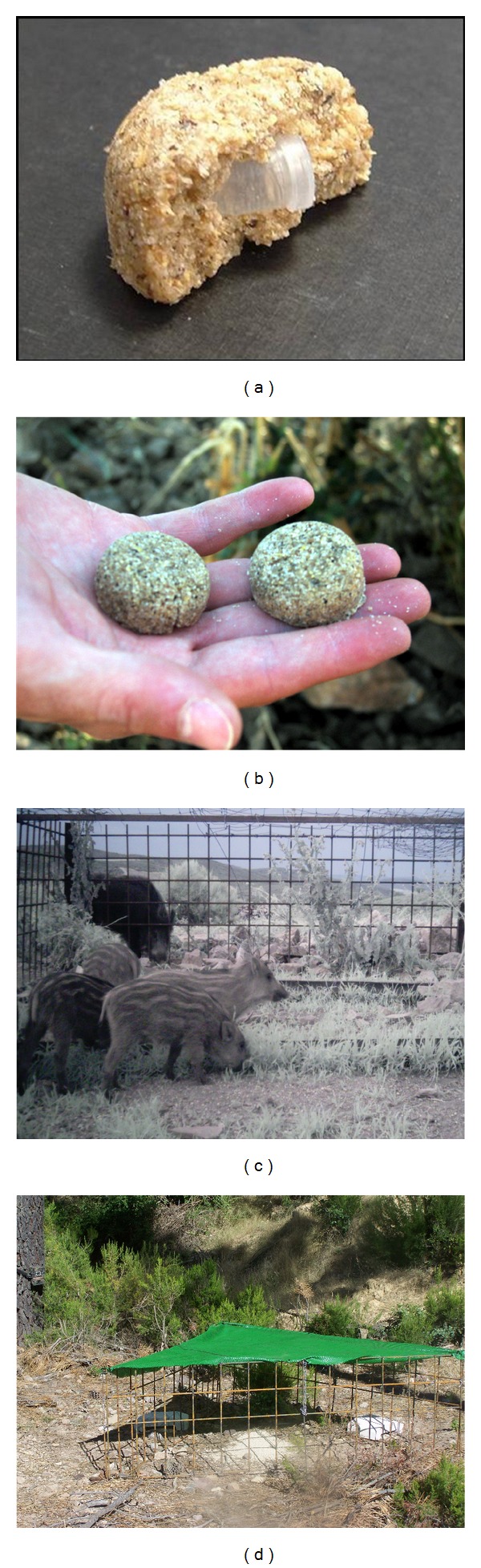
(a) and (b) Baits developed to deliver vaccines to wild boar in Spain. They are composed of piglet feed, paraffin, sucrose and cinnamon-truffle powder attractant. (c) Wild boar piglets consuming baits inside selective feeders. (d) Portable selective feeder used to deliver baits to young wild boar.

Bait consumption rate and host specificity depend directly on the delivery method employed [10, 55]. To date, three delivery systems have been designed to allow free-ranging *Sus scrofa* access to baits while preventing bait consumption by nontarget species: the Boar-Operated-System [64–66], the HogHopper [67, 68], and portable selective wild boar piglet feeders of Spanish patent [37]. The BOS consists of a metal pole onto which a round perforated base is attached. A metal cone with wide rim slides up and down the pole and fully encloses the base onto which the baits are placed. This system has been tested in United Kingdom [66] and the United States [65] showing low bait consumption by nontarget species. However, a possible disadvantage to this system is its cost [65]. The Hog-Hopper is a new box-shaped bait delivery device designed to allow feral pigs to access poison baits in the station, and to restrict other species (such as Australian native species and livestock) from taking bait. The door of the device is easily raised by feral pigs allowing them to feed on bait, but it excludes nontarget species that lack the physical attributes to lift the sliding door. Small rodents are also unable to access baits. The device has the added benefit of preventing bait from being exposed to rain, thereby preventing bait degradation [67, 68]. Selective feeders were used by Ballesteros et al. [37] in order to reduce bait consumption by nontarget species in southern Spain. These triangular-shaped feeders (side = 1 m) consist of a 1-cm-*∅* metal-grid cage with an opening (15-cm wide) to allow only access of young wild boar (Figure 1(c)). A green mesh that provides shade covers the cage (Figure 1(d)). Although this system was found to be highly selective for wild boar piglets [10], occasionally small animals such as badgers can enter inside the feeders and have access to the baits [37].

The success of vaccination programs is also determined by the timing of bait delivery. For example, early summer would be the best timing for TB vaccine bait delivery to wild boar piglets in south-central Spain [10]. In addition, bait consumption by wild boar or feral pigs is better if the prebaiting period lasts longer (e.g., feeding corn weekly for three weeks) so that animals get accustomed to feed in the place where baits will be delivered [37, 69, 70]. Other factors such as baiting and/or free-ranging *Sus scrofa* densities can affect bait consumption by target species [37]. Ballesteros et al. [37] found marked-bait consumption by up to 73% wild boar piglets at a bait density of 30 baits/km^2^ and using one piglet feeder per 2 km^2^. These baiting densities were lower than those used in previous studies in other countries (e.g., 68 to 489 baits/km^2^ [41, 57]. Therefore, in future TB vaccination experiments, it would be desirable to use higher baiting densities to target a higher percentage of the wild boar population. 

## 5. Tuberculosis Vaccines in Wildlife

Live vaccines are believed to confer more protection against mycobacterial infections than killed vaccines [71]. This is the case of the attenuated live strain of *M. bovis *BCG [72], which is currently the only vaccine approved for vaccination of humans against TB [73, 74]. Since 1921, BCG has been used worldwide and reports of adverse reactions arising from the use of this vaccine have been relatively uncommon [75]. Also, BCG is the most widely used vaccine for TB control in wildlife reservoirs. Experiments in controlled environments have been carried out in several host species such as badgers [76], brushtail possums [77], Cape buffalo (*Syncerus caffer*) [78], white-tailed deer [54, 79], wild boar [38], and ferrets (*Mustela furo*) [80]. In addition, recent reports on field vaccination of badgers in the UK [81] and possums in New Zealand [53] encourage the use of BCG for TB control in wildlife. Additionally, due to its long and widespread use in different species, licensing BCG for field use in wild animals is easier than licensing a newly developed vaccine [81, 82].

However, the use of BCG has some disadvantages, including (1) its variable efficacy in humans and cattle due to differences among BCG vaccine strains, trial methodology, and prior host sensitization to a variety of environmental mycobacteria [71, 83–87], (2) the possibility to cause disease or to infect nontarget individuals and cause interference with TB diagnosis [88–92], and (3) its limited half life in the environment and during vaccine preparation, shipment, or storage [93].

The use of killed vaccines would eliminate the risk of causing TB and should limit the likelihood of diagnostic interference and make field vaccination protocols cheaper. Several authors have found experimental evidence indicating that nonviable bacilli are able to produce some degree of protection to TB in guinea pigs [94–99], mice [100–103] and dogs [104]. In wildlife, only limited information exists regarding inactivated vaccines. Experiments with inactivated vaccines have been conducted in deer, brushtail possums, and recently in wild boar [105–107]. Deer were vaccinated with two doses of heat-killed BCG (5 × 10^7^ cfu) in an oil adjuvant finding no protection against experimental challenge with virulent *M. bovis *[105]. In brushtail possums, the heat-inactivated *M. vaccae* was used to improve the effectiveness of live BCG to protect against bTB [106]. Recently, heat-inactivated *M. bovis* was found to confer protection against TB similar to BCG to wild boar [107].

## 6. Wild Boar Response to BCG Vaccination 

The first results about wild boar response to BCG vaccination were documented in 2009, after vaccinating seven animals by the intramuscular route [108]. Later, a subsequent study by Ballesteros et al. [38] analyzed wild boar responses to oral BCG vaccination and challenge with a *M. bovis* field strain. Purpose-designed oral baits were used for the experiment [36]. The oropharyngeal route was found appropriate for wild boar experimental infection since lesions recorded resembled those of natural mycobacterial infections [109]. This research allowed defining the infection model and a lesion scoring system for wild boar TB [38], while further experiments increased the information on wild boar response to BCG vaccination [107]. 

In captive wild boar, BCG has shown significant levels of protection against challenge with a virulent *M. bovis* field strain. Culture scores and lesion scores of orally BCG-vaccinated wild boar were consistently lower in vaccinated than in control nonvaccinated animals [38, 107]. In addition, the reduction of the lesion and culture scores in the thoracic organs has been between 67% and 90% as compared to unvaccinated controls [107]. Vaccinated wild boar exposed to low-or-medium doses of *M. bovis* (10^2^ cfu or 10^4^ cfu) by the oropharyngeal route generally remain either uninfected or develop only limited lesions [38].

Antibody responses of wild boar against *M. bovis* have been detected reliably with specific serologic tests [21, 110, 111]. This enzyme-linked immunosorbent assay uses *M. bovis* purified protein derivative (bPPD ELISA test). Antibodies to bPPD increase only slightly and late after challenge and correlate with the total lesion scores of BCG vaccinated and *M. bovis *challenged wild boar [107]. Additionally, an innovative dual-path platform test (DPP test) using MPB83 and CFP10/ESAT-6 antigens has also been used to monitor antibody production in vaccination experiments in captive wild boar [107, 111]. Also, IFN-gamma production in response to bPPD has been detected in both BCG vaccinated and unvaccinated wild boar after *M. bovis* challenge [107]. 

## 7. Wild Boar Response to Vaccination with Heat-Inactivated *M. bovis*

Recently, a heat-killed *M. bovis* vaccine for oral and parenteral use was developed and tested in wild boar [107]. Each oral dose contained 6 × 10^6^ bacteria in 2 mL of PBS, and each parenteral dose contained the same number of bacteria in 1 mL Montanide ISA 50 V (Seppic, Castres, France). 

The first study incorporating this new inactivated vaccine showed that oral or parenteral vaccination with heat-inactivated *M. bovis* conferred a similar protection after challenge when compared to oral vaccination with BCG, and that the response of wild boar to both vaccines was similar. Although a high challenge dose was used (10^6^ cfu), this vaccination protocol reduced the number and severity of lesions and the infection burden, particularly in the thoracic region [107]. 

The dynamics of antibody production, IFN-gamma response, and gene expression were similar in oral BCG- and inactivated *M. bovis*-vaccinated animals. Wild boar parenterally vaccinated with the inactivated vaccine responded to the MPB83 antigen but not to bPPD immediately after vaccination, suggesting potential use of these ELISAs to distinguish between parenterally vaccinated and exposed wild boar [107].

## 8. Wild Boar-Pathogen Interactions and Protection against TB

The study of host-pathogen interactions allowed identifying biomarkers of resistance/susceptibility to tuberculosis in wild boar and using these biomarkers for vaccine development [63, 107, 108, 112–115]. The expressions of some of these genes such as complement component 3 (C3) and methylmalonyl coenzyme A mutase (MUT) were shown to correlate with resistance to natural *M. bovis* infection and protection against *M. bovis* challenge in BCG-vaccinated wild boar [9, 38, 107, 108, 116]. In these experiments, C3 and/or MUT mRNA levels were higher in nontuberculous than in tuberculous adult wild boar naturally exposed to mycobacterial infection, decreased after *M. bovis* infection and increased with BCG vaccination, with higher mRNA levels in protected animals [9, 38, 107, 108, 113, 114, 116, 117]. Additionally, MUT may be genetically associated with resistance to tuberculosis in wild boar [113, 116, 117]. 

The mechanisms by which C3 and MUT expression contributes to resistance to mycobacterial infection remain unknown. The complement system has been shown to be involved in mycobacterial pathogenesis and *M. tuberculosis* activates the alternative pathway of complement and binds C3 protein, resulting in enhanced phagocytosis by complement receptors (CR3) on human alveolar macrophages [118, 119]. A similar mechanism may occur with *M. bovis* in which C3 opsonophagocytosis of mycobacteria by macrophages may result in the inhibition of host bactericidal responses and pathogen survival [118]. Consequently, higher levels of C3 in wild boar may allow increased binding of C3 to CR3 to promote phagocytosis and effective killing of bacteria, while interfering with CR3-mediated opsonic and nonopsonic phagocytosis of mycobacteria [114]. For MUT, a hypothesis was recently discussed to suggest that host genetically defined higher MUT expression levels result in lower serum cholesterol concentration and tissue deposits that increase the protective immune response to *M. bovis*, thus resulting in resistance to tuberculosis and better response to BCG vaccination [117]. 

The mechanism of protection from BCG vaccination involves a reduction of the haematogenous spread of mycobacteria from the site of primary infection. It protects against the acute manifestations of the disease, and reduces the lifelong risk of endogenous reactivation and dissemination associated with foci acquired from prior infection [120]. 

It is tempting to speculate that BCG protection in wild boar would involve distinct systemic and mucosal populations of effector memory T cells. Immune genes with significant overexpression in nontuberculous than in tuberculous adult wild boar naturally exposed to mycobacterial infection include RANTES (aka Chemokine (C-C motif) ligand 5; CCL5), IFN-gamma, and IL4 [108]. The mRNA levels of these genes also increased after parenteral and oral BCG vaccination of wild boar [38, 108], thus suggesting that IFN-gamma and activated RANTES-secreting CD8 (+) and/or CD4 (+) T lymphocytes may be key players in BCG-induced protective response in wild boar. However, although it is generally recognized that humoral immunity is not important for the control of tuberculosis [121], IL4-induced antibody response against *M. bovis* may be important for tuberculosis control in wild boar. IL4 overexpression in nontuberculous and BCG-vaccinated wild boar suggests that antibodies against mycobacterial proteins may be used for disease surveillance and treatment monitoring in this species [21, 107, 110, 111] and underline the existence of host-specific responses to mycobacterial infection [122] as the increase in IL4 levels correlates with disease severity in humans [123] but not in wild boar.

Inactivated vaccines stimulate specific CD4 (+) cell populations that recognize the antigen versus a live vaccine that stimulates many T cell populations simultaneously. While antibody and IFN-gamma responses increased after vaccination in parentally BCG-vaccinated wild boar, in orally BCG and inactivated *M. bovis* vaccinated wild boar, only MUT mRNA levels correlated with protection [107]. These results are difficult to explain before further experiments help to characterize the mechanism by which vaccination with the inactivated vaccine protects against tuberculosis in wild boar. Taken together, these results suggest different protective mechanisms between parenteral and oral inactivated mycobacterial vaccines and, at least for MUT, expression levels could be a marker of protection against tuberculosis and may be used to characterize host response to BCG vaccination in wild boar.

## 9. Wild Boar Vaccination Biosafety Issues 

Four main biosafety issues must be considered before delivery of oral baits containing live vaccines such as BCG to wild boar: (1) potential effects of high vaccine doses (e.g. ten times the normal dose) on wild boar health; (2) potential survival of *M. bovis* BCG in vaccinated wild boar; (3) potential excretion of *M. bovis* BCG by vaccinated wild boar; (4) vaccine-containing bait uptake by nontarget species, particularly by cattle. 

Regarding point (1), it is highly important to determine that high doses of vaccine do not affect the animal's health since it is likely that few individuals can gain access to a high number of baits during field vaccination campaigns. In the case of wild boar, no adverse effects that can be attributed to the vaccine have been detected in vaccinated individuals [38, 107, 108]. Moreover, wild boar treated with high vaccine doses of up to 3.0 × 10^6^ cfu did not show any adverse effect after BCG administration [38]. 

Concerning point (2), *M. bovis* BCG has never been isolated from tissues of vaccinated wild boar, despite the occasionally high doses used (the authors, unpublished information). However, in other species, such as brushtail possum and deer, BCG was isolated in tissues of oral BCG vaccinated animals after necropsy [124, 125]. 

Regarding point (3), the potential of faeces from vaccinated wild boar to lead to the accidental exposure of non-target species to BCG, shedding of BCG following bait ingestion has been tested under laboratory conditions over a period of seven days post vaccination. The analyses yielded no BCG isolates (unpublished data). In other TB hosts, BCG is detected in faeces only for a short period of time after ingestion [52, 125]. 

Finally, point (4), it is necessary to consider the possibility of bait consumption by nontarget species. Oral baits developed by Ballesteros et al. [36] were found highly palatable to both wild and domestic animals [55]. This fact could have negative effects in areas where cattle and wild reservoirs coexist since accidental consumption of BCG-containing baits by cattle could interfere in the TB test and slaughter campaigns. However, this risk can be reduced by using deployment strategies that assure that only target species gain access to bait such as selective feeders [10, 37]. Moreover, the scheduled preliminary field experiments are taking place in sites without cattle [37]. In addition, the research towards the development of BCG-specific blood tests for cattle, and the relatively short duration of BCG-induced reactivity in livestock contribute to limit the concerns [126].

Further information regarding BCG biosafety is needed to satisfy regulatory and licensing requirements for release of oral bait vaccines to wildlife [82, 125]. Therefore, new laboratory experiments will be conducted in order to assess the potential oral or nasal excretion of BCG by vaccinated wild boar. In addition, cattle will be exposed to BCG-containing baits under controlled conditions to assess the likeliness of developing a positive skin test. Other important information regarding biosafety will be derived from the first controlled field experiments starting soon in Southern Spain. Furthermore, the recent development of an inactivated *M. bovis* vaccine would significantly reduce the safety issues, since no viable organisms are used [107].

## 10. Conclusions and Future Research Directions

During the last decade, research on TB epidemiology and oral vaccine development and characterization in wild boar allowed considering oral vaccination among available TB control tools. The continued applied and basic research on integrated TB control at the wildlife-livestock interface will hopefully yield even more significant advances in the future. 

**Figure 2:**
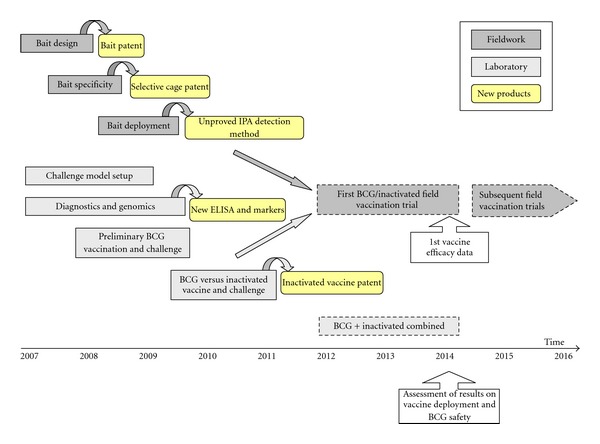
Flowchart of the laboratory and field research and key results towards oral vaccination of Eurasian wild boar against *Mycobacterium bovis*. Dashed boxes indicate future experiments.

Future research on TB vaccination in wild boar will include both new controlled laboratory and field experiments (Figure 2). Results obtained in experiments comparing the efficacy of inactivated *M. bovis* and BCG vaccines encourage testing combinations of these vaccine preparations. The characterization of the immune mechanisms that support protection against tuberculosis after vaccination with BCG and inactivated vaccines are essential to advance in the development of new improved vaccines and/or vaccination schemes.

 A controlled and replicated experimental oral vaccination trial will start soon in southern Spain. The goals of this first field trial are assessing the response of wild boar to oral BCG and heat-killed *M. bovis* vaccination under field conditions, gathering information on safety aspects and analyzing the cost-effectiveness of vaccination for TB control in wild boar. This includes modeling the outcome of vaccination as compared to population control. 

Regarding models, preliminary data gathered from individual-based models suggest that vaccinating piglets over a long-term period has the potential to successfully eradicate bTB from wild boar reservoirs in southern Spain. Further research into the transmission rates between bTB hosts and the efficacy of the vaccine itself, but also on the cost-effectiveness of wild boar vaccination as compared to population control (and their combinations) will add important reinforcements to these initial findings. 

## Figures and Tables

**Table 1:** Characteristics of commercial, registered, or patented baits designed for the delivery of vaccines or pharmaceuticals to wild boar and feral pigs. Disadvantages include particularly their suitability for vaccine delivery to piglets.

Bait	Shape and size	Use	Advantages	Disadvantages	References
Riemser	Square shape (4 × 4 × 1.5 cm)	Delivery of vaccines against classical swine fever in Europe	Resistant to water and moisture	Not resistant to warm temperatures Not completely consumed by <4.5 month-old wild boar piglets	[33–35]

IREC, Spain	Hemispherical shape ( *Ø*3.4 × 1.6 cm; Figures 1(a) and 1(b))	Delivery of vaccines against *Mycobacterium bovis* in Spain	Resistant to high temperatures Well accepted by 2–4 month-old wild boar piglets	Not resistant to water and moisture	[10, 36–38]

PIGOUT	Cylindrical shape (9 × 5 cm)	Delivery of toxicants or pharmaceuticals to feral pigs in Australia and USA	Resistant to high temperatures	Large size would not be suitable for piglets	[39–42]
